# Supplementary material for: How neuronal morphology impacts the synchronisation state of neuronal networks
Source: PLoS Comput Biol. 2024 Mar 4;20(3):e1011874. doi: 10.1371/journal.pcbi.1011874 (PMC10939433; doi:10.1371/journal.pcbi.1011874)
Supplement: S1 Text — (PDF) [file pcbi.1011874.s001.pdf]

How neuronal morphology impacts the synchronisation state of neuronal networks - S1 Text

Robert P Gowers[1,2,*], Susanne Schreiber[1,2,*]

[1] Institute for Theoretical Biology, Humboldt-University of Berlin, Berlin, Germany
[2] Bernstein Center for Computational Neuroscience, Berlin, Germany

*** For correspondence:** robert.gowers@hu-berlin.de and s.schreiber@hu-berlin.de

Equations and Parameters of the Morris-Lecar Model

The single-compartment Morris-Lecar voltage equation is

$$f_{\mathrm{S}}(n, v_\sigma) = \frac{1}{C_\sigma}\left[G_\sigma(E_L - v_\sigma) + m_\infty(v_\sigma)G_{\mathrm{Ca}}(E_{\mathrm{Ca}} - v_\sigma) + nG_{\mathrm{K}}(E_K - v_\sigma) + I_{\mathrm{ext}}\right], \tag{A}$$

while the dynamics of the recovery variable n are given by

$$\frac{\mathrm{d}n}{\mathrm{d}t} = \frac{n_\infty(v_\sigma) - n}{\tau_n(v_\sigma)}, \quad \tau_n(v_\sigma) = \left[\phi\cosh\left(\frac{v_\sigma - A_{\mathrm{K}}}{4\Delta_{\mathrm{K}}}\right)\right]^{-1}. \tag{B}$$

Steady-state values of the activation variables are given in terms of sigmoidal functions of v_σ

$$m_\infty(v_\sigma) = \left[1 + \exp\left(-\frac{v_\sigma - A_{\mathrm{Ca}}}{\Delta_{\mathrm{Ca}}}\right)\right]^{-1}, \quad n_\infty(v_\sigma) = \left[1 + \exp\left(-\frac{v_\sigma - A_{\mathrm{K}}}{\Delta_{\mathrm{K}}}\right)\right]^{-1} \tag{C}$$

Parameter	Value	Units
G_σ	2	nS
C_σ	20	pF
E_L	-60	mV
G_{Ca}	4	nS
E_{Ca}	120	mV
A_{Ca}	-1.2	mV
Δ_{Ca}	9	mV
G_{K}	8	nS
E_{K}	-80	mV
A_{K}	12	mV
Δ_{K}	8.7	mV
ϕ	1/15	ms^{-1}

Table A. Default parameters used in this paper for the Morris-Lecar model, taken from [1] and scaled by a somatic area of 100 $\mu\mathrm{m}^2$.

Relative Conductance Formulation

The somatic equation in terms of absolute conductances and capacitances

$$C_\sigma \frac{\mathrm{d}v_\sigma}{\mathrm{d}t} = G_\sigma(E_L - v_\sigma) + \sum_{j=1}^{B} I_{a,j}(\boldsymbol{a}_j, v_\sigma) + I_{\mathrm{ext}} + \rho G_\sigma \lambda \left.\frac{\partial v_\delta}{\partial x}\right|_{x=0}, \tag{D}$$

can be transformed into an equation in terms of a passive time constant τ_σ and potentials $\mu_{a,j}$, μ_{ext}

$$\tau_\sigma \frac{\mathrm{d}v_\sigma}{\mathrm{d}t} = E_L - v_\sigma + \sum_{j=1}^{B} \mu_{a,j}(\boldsymbol{a}_j, v_\sigma) + \mu_{\text{ext}} + \rho\lambda \left.\frac{\partial v_\delta}{\partial x}\right|_{x=0}. \tag{E}$$

Each active potential is given in terms of its relative conductance $h_j = G_j/G_\sigma$

$$\mu_{a,j}(\boldsymbol{a}_j, v) = h_j(E_j - v) \prod_{i\in \boldsymbol{a}_j} a_i(v)^{p_i}. \tag{F}$$

For a fixed somatic conductance G_σ, local bifurcations can be calculated in this system with μ_{ext} and ρ as bifurcation parameters rather than I_{ext} and G_{in}.

Active Dendrite Simulations

While theoretical analysis of a fully active dendrite cannot be performed using the methods in this paper, we can extract the onset PRCs via simulation. In this case, we add the same active conductances to the dendrite as at the soma. Denoting each active current density as $J_{\delta,j}$, we start from the active cable equation

$$c_\delta \frac{\partial v_\delta}{\partial t} = g_\delta(E_L - v_\delta) + \sum_{j=1}^{B} J_{\delta,j}(\boldsymbol{a}_j(x), v_\delta) + g_\delta \lambda^2 \frac{\partial^2 v_\delta}{\partial x^2}, \tag{G}$$

and then we divide by the leak conductance density g_δ

$$\tau_\delta \frac{\partial v_\delta}{\partial t} = E_L - v_\delta + \sum_{j=1}^{B} \mu_{\delta,j}(\boldsymbol{a}_j(x), v_\delta) + \lambda^2 \frac{\partial^2 v_\delta}{\partial x^2}, \tag{H}$$

where each dendritic active potential is given in terms of a relative conductance density $h_{\delta,j}$

$$\mu_{\delta,j}(\boldsymbol{a}_j(x), v) = h_{\delta,j}(E_j - v) \prod_{i\in \boldsymbol{a}_j} a_i(v)^{p_i}. \tag{I}$$

Here we will make each dendritic relative conductance a fixed fraction ϵ of its corresponding somatic relative conductance $h_{\delta,j} = \epsilon h_j$, where ϵ is the same across the channels. This means that the active cable equation is

$$\tau_\delta \frac{\partial v_\delta}{\partial t} = E_L - v_\delta + \epsilon \sum_{j=1}^{B} \mu_{a,j}(\boldsymbol{a}_j(x), v_\delta) + \lambda^2 \frac{\partial^2 v_\delta}{\partial x^2}. \tag{J}$$

The limit $\epsilon = 0$ corresponds to the passive dendrite model analysed in this study, while when $\epsilon = 1$ the relative conductance densities in the dendrite and soma are identical.

We compare the PRCs at onset for $\epsilon = 0$ (passive), $\epsilon = 0.1$ (weakly active) and $\epsilon = 1$ (identically active), as shown in Fig A. We see that at low G_{in}, the PRCs are all of the symmetric SNIC type. For $\epsilon = 0.1$, increasing G_{in} deforms the PRC in qualitatively the same manner as the passive case, first becoming asymmetric for $G_{\text{in}} = 5.5$ nS and then adopting a negative phase region at $G_{\text{in}} = 5.9$ nS. We note that the PRCs are not quantitatively identical to the passive dendrite model however, with the asymmetic HOM PRC (Fig Aiii) having a broader peak and a different peak location for the subcritical Hopf PRC (Fig Aiv). The adoption of the asymmetric HOM PRC also seems to occur at higher G_{in}, with the PRC at $G_{\text{in}} = 5.4$ being only weakly asymmetric.

For the identically active case of $\epsilon = 1.0$, the PRC remains unchanged across all input conductances tested. This is intriguing, but makes sense because while the value of I_{ext} required to induce spiking increases with increasing G_{in}, the relative conductance contributions do not as the channel density of all the conductances is the same everywhere. Unlike the passive or weakly-active cases, the overall conductance portfolio is not diluted by a stronger influence of passive channels from increasing G_{in}.

Overall, the active dendrite PRCs show that provided the channel densities in the dendrite and soma differ, as is typically the case, the relative weight of the dendrite can shift the dynamical spiking type of the neuron. In the large dendrite limit, the onset dynamics of the neuron tend towards the onset dynamics of the dendrite itself. Hence the large dendrite limit of the passive dendrite is one without spiking (excitation block) and the onset type of the identically active dendrite remains the same. Provided that the dendrite and soma have quantitatively different dynamics, the PRC will be affected by changes to the relative dendritic size.

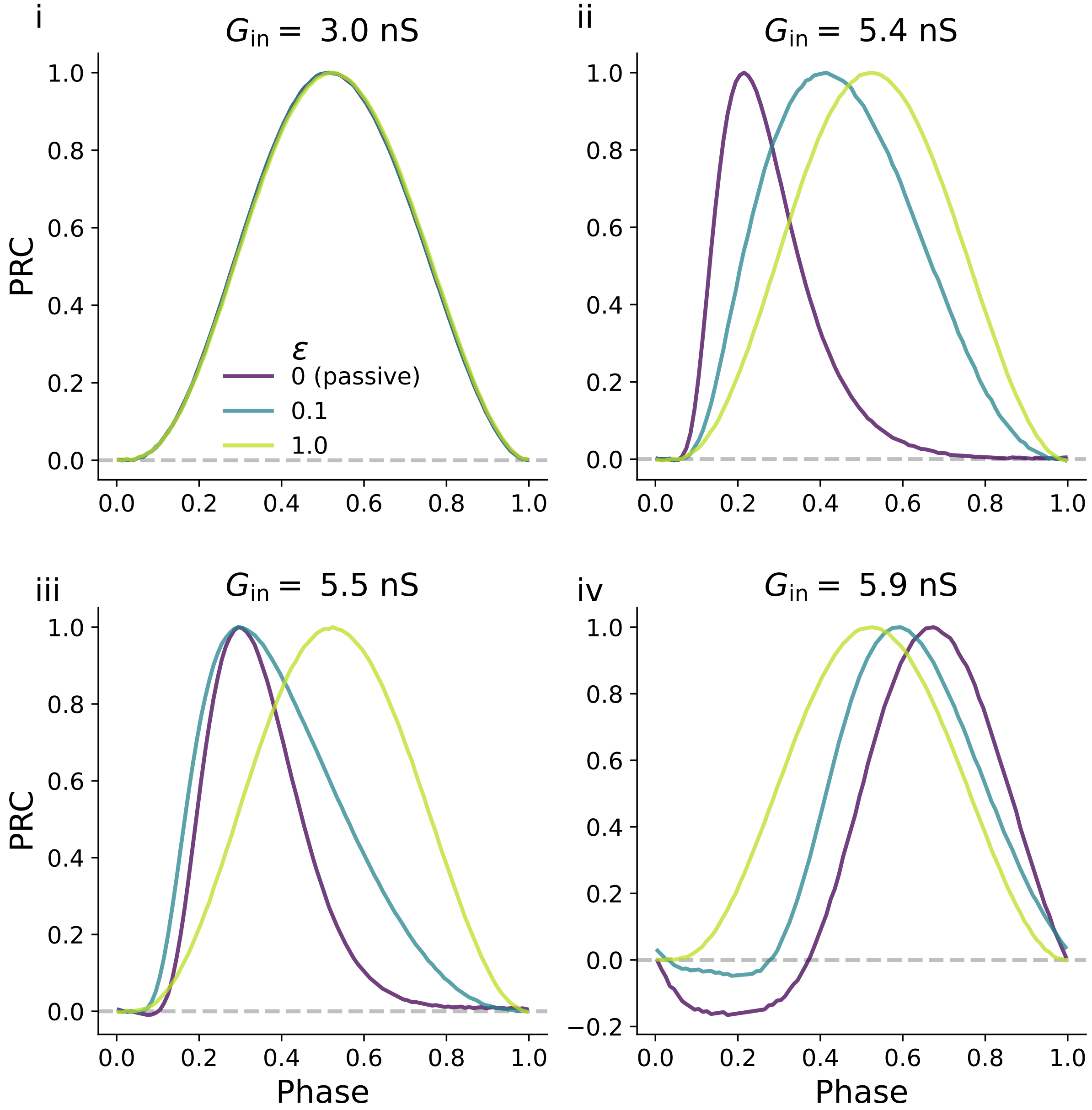

Fig A. The PRC of the active dendrite-and-soma model can change with increased dendritic conductance load depending on the relative strength of the active channel conductances. For $\epsilon = 0.1$, the PRC starts symmetric (i) before becoming more asymmetric (ii), adopting a HOM-like PRC (iii), and then having a negative phase region (iv). When $\epsilon = 1.0$, the relative channel conductance densities are uniform across the neuron and the PRC remains unchanged with G_{in}. For all three cases $\tau_\delta = 10$ ms.

Derivation of the Local Bifurcation Equations

The Method of Lines

For finite-dimensional dynamical systems, such as conductance-based point-neuron models, the Jacobian of the system has a finite number of eigenvalues. This gives a clear, but by no means trivial, approach to calculating the Hopf, BT and BTC bifurcations [2, 3, 4]. However, for a spatially continuous system, the number of dimensions is not finite. Fortunately, by using the method lines, we can derive equations for the bifurcations in an analogous fashion.

The method of lines discretises a spatially continuous system by dividing the cable into M compartments with index $k = 1, .., M$ [5]. In this case, we will use a constant spatial step size Δx such that $v_\delta(k\Delta x, t) = v_k(t)$ and use the following discretised approximations of the first and second-order spatial derivatives

$$\frac{\partial^2 v_k}{\partial x^2} \approx \frac{v_{k+1} - 2v_k + v_{k-1}}{\Delta x^2}, \quad \frac{\partial v_k}{\partial x} \approx \frac{v_{k+1} - v_k}{\Delta x}. \tag{K}$$

Note that $v_{k=0} = v_\sigma$ and that we must incorporate the sealed-end boundary condition, which changes the second derivative approximation at the distal dendritic end to

$$\frac{\partial^2 v_M}{\partial x^2} \approx \frac{2v_{M-1} - 2v_M}{\Delta x^2}. \tag{L}$$

This discretisation means that we now have a $M + K + 1$ dimensional dynamical system, where we recall that K is the number of active variables. The active variable equations remain unchanged from (14), while the voltage equations in the discretised system are

$$\begin{aligned}
\frac{\mathrm{d}v_\sigma}{\mathrm{d}t} &= f_\mathrm{S}(\boldsymbol{a}, v_\sigma) + \frac{\rho G_\sigma \lambda}{C_\sigma \Delta x}(v_1 - v_\sigma), \\
\frac{\mathrm{d}v_k}{\mathrm{d}t} &= \frac{E_L - v_k}{\tau_\delta} + \lambda^2 \frac{v_{k+1} - 2v_k + v_{k-1}}{\Delta x^2 \tau_\delta}, \quad k = 1, ..., M-1 \\
\frac{\mathrm{d}v_M}{\mathrm{d}t} &= \frac{E_L - v_M}{\tau_\delta} + \lambda^2 \frac{2v_{M-1} - 2v_M}{\Delta x^2 \tau_\delta}.
\end{aligned} \tag{M}$$

From Eqs 14 and M we can obtain the Jacobian $\boldsymbol{J}$ for the discretised system, allowing us to approach the problem of calculating the local bifurcations in a similar manner to the point-neuron model. Although the Jacobian now has $(M + K + 1) \times (M + K + 1)$ elements, most of these are zero, and those associated with the dendritic equations do not depend on the state variables. Further details of this approach are given in the Appendix, but it is sufficient here to say that the method of lines allows local bifurcations to be calculated for the *discretised* dendrite-and-soma model. With the equations from the discretised dendrite, we can go further and take the continuum limit ($\Delta x \to 0$) to yield equations for the spatially *continuous* DS model. It is these spatially continuous bifurcation values which we show in the bifurcation diagrams.

With the method of lines established, we can now describe how to calculate the Hopf, BT and BTC bifurcations in a spatially continuous DS model.

Discretised Jacobian Matrix

Upon discretising the dendrite into M compartments each of width Δx, we have the following $M + K + 1$ differential equations for the system

$$\frac{\mathrm{d}a_i}{\mathrm{d}t} = f_{ai}(a_i, v_\sigma) = \frac{a_{i,\infty}(v_\sigma) - a_i}{\tau_i(v_\sigma)}, \quad i = 1, ..., K, \tag{N}$$

$$\frac{\mathrm{d}v_\sigma}{\mathrm{d}t} = f_\sigma(\boldsymbol{a}, \boldsymbol{v}) = f_\mathrm{S}(\boldsymbol{a}, v_\sigma) + \frac{\lambda \rho G_\sigma}{\Delta x C_\sigma}(v_1 - v_\sigma), \tag{O}$$

$$\frac{\mathrm{d}v_k}{\mathrm{d}t} = f_k(\boldsymbol{v}) = \frac{E_L - v_k}{\tau_\delta} + \frac{\lambda^2}{\Delta x^2 \tau_\delta}(v_{k-1} - 2v_k + v_{k+1}), \quad k = 1, ..., M-1 \tag{P}$$

$$\frac{\mathrm{d}v_M}{\mathrm{d}t} = f_M(\boldsymbol{v}) = \frac{E_L - v_M}{\tau_\delta} + \frac{\lambda^2}{\Delta x^2 \tau_\delta}(2v_{M-1} - 2v_M). \tag{Q}$$

This allows us to write the Jacobian matrix as

$$\boldsymbol{J} = \begin{pmatrix} \frac{\partial f_{a1}}{\partial a_1} & \cdots & \frac{\partial f_{a1}}{\partial a_K} & \frac{\partial f_{a1}}{\partial v_\sigma} & \frac{\partial f_{a1}}{\partial v_1} & \cdots & \frac{\partial f_{a1}}{\partial v_M} \\ \cdots & \cdots & \cdots & \cdots & \cdots & \cdots & \cdots \\ \frac{\partial f_\sigma}{\partial a_1} & \cdots & \frac{\partial f_\sigma}{\partial a_K} & \frac{\partial f_\sigma}{\partial v_\sigma} & \frac{\partial f_\sigma}{\partial v_1} & \cdots & \frac{\partial f_\sigma}{\partial v_M} \\ \frac{\partial f_1}{\partial a_1} & \cdots & \frac{\partial f_1}{\partial a_K} & \frac{\partial f_1}{\partial v_\sigma} & \frac{\partial f_1}{\partial v_1} & \cdots & \frac{\partial f_1}{\partial f_M} \\ \cdots & \cdots & \cdots & \cdots & \cdots & \cdots & \cdots \\ \frac{\partial f_M}{\partial a_1} & \cdots & \frac{\partial f_M}{\partial a_K} & \frac{\partial f_M}{\partial v_\sigma} & \frac{\partial f_M}{\partial f_1} & \cdots & \frac{\partial f_M}{\partial v_M} \end{pmatrix}, \tag{R}$$

which we will split into four block matrices

$$\boldsymbol{J} = \begin{pmatrix} \mathcal{A} & \mathcal{B} \\ \mathcal{C} & \mathcal{D} \end{pmatrix}, \quad \mathcal{A}_{ij} = \frac{\partial f_{ai}}{\partial a_j}, \quad i,j = 1,...,K, \quad \mathcal{B}_{ij} = \frac{\partial f_{ai}}{\partial v_j}, \quad i = 1,...,K,\ j = \sigma, 1, ..., M,$$
$$\mathcal{C}_{ij} = \frac{\partial f_i}{\partial a_j}, \quad i = \sigma, 1, ..., M,\ j = 1,...,K, \quad \mathcal{D}_{ij} = \frac{\partial f_i}{\partial v_j}, \quad i = \sigma, 1, ..., M,\ j = \sigma, 1, ..., M. \tag{S}$$

For the partial derivatives of f_{ai} we have

$$\frac{\partial f_{ai}}{\partial a_j} = \begin{cases} -\frac{1}{\tau_i(v_\sigma)}, & i = j \\ 0, & i \neq j \end{cases}, \tag{T}$$

$$\frac{\partial f_{ai}}{\partial v_\sigma} = \frac{1}{\tau_i}\frac{\mathrm{d}a_{i,\infty}}{\mathrm{d}v_\sigma} + (a_{i,\infty} - a_i)\frac{\partial}{\partial v_\sigma}\left(\frac{1}{\tau_i}\right), \tag{U}$$

where $\partial f_{ai}/\partial v_\sigma = \frac{1}{\tau_i}\mathrm{d}a_{i,\infty}/\mathrm{d}v_\sigma$ when evaluated at equilibrium. Since the active variables depend only on the somatic voltage, $\partial f_{ai}/\partial v_k = 0$. In terms of the block matrices, this means that $\mathcal{A}$ is a diagonal matrix with

$$\mathcal{A}_{ii} = -\frac{1}{\tau_i}, \quad i = 1, ..., K, \tag{V}$$

while the matrix $\mathcal{B}$ only has one non-zero column

$$\mathcal{B}_{i\sigma} = \frac{1}{\tau_i}\frac{\mathrm{d}a_{i,\infty}}{\mathrm{d}v_\sigma}, \quad B_{ij} = 0,\ j \neq \sigma. \tag{W}$$

For the somatic voltage equation, we define $\Lambda = \lambda^2/\Delta x^2$ and write the voltage derivatives as

$$\frac{\partial f_\sigma}{\partial v_\sigma} = \frac{\partial f_\mathrm{S}}{\partial v_\sigma} - \frac{\sqrt{\Lambda}\rho G_\sigma}{C_\sigma}, \quad \frac{\partial f_\sigma}{\partial v_1} = \frac{\sqrt{\Lambda}\rho G_\sigma}{C_\sigma}, \quad \frac{\partial f_\sigma}{\partial v_k} = 0, \quad k > 1. \tag{X}$$

Turning to the dendritic equations, since the dendrite is passive $\partial f_k/\partial a_i = 0$ for all i, k. For the voltage derivatives

$$\frac{\partial f_1}{\partial v_\sigma} = \frac{\Lambda}{\tau_\delta}, \quad \frac{\partial f_k}{\partial v_\sigma} = 0, \quad k \neq 1, \quad \frac{\partial f_k}{\partial v_j} = \begin{cases} \Lambda/\tau_\delta, \quad j = k \pm 1,\ k \neq M \\ -(1 + 2\Lambda)/\tau_\delta, \quad j = k \\ 0, \quad \text{otherwise} \end{cases}, \quad \frac{\partial f_M}{\partial v_{M-1}} = 2\Lambda/\tau_\delta, \tag{Y}$$

which means that the matrix $\mathcal{C}$ only has one non-zero row

$$\mathcal{C}_{\sigma j} = \frac{\partial f_\sigma}{\partial a_j}, \quad C_{ij} = 0, \quad i \neq \sigma, \tag{Z}$$

while $\mathcal{D}$ is a tridiagonal matrix

$$\mathcal{D}_{\sigma\sigma} = \frac{\partial f_{\mathrm{S}}}{\partial v_\sigma} - \frac{\sqrt{\Lambda}\rho G_\sigma}{C_\sigma}, \quad D_{\sigma 1} = \frac{\sqrt{\Lambda}\rho G_\sigma}{C_\sigma},$$
$$\mathcal{D}_{i,i\pm1} = \frac{\Lambda}{\tau_\delta}, \quad \mathcal{D}_{ii} = -\frac{(1+2\Lambda)}{\tau_\delta}, \quad i = 1, ..., M-1, \quad \mathcal{D}_{M,M-1} = \frac{2\Lambda}{\tau_\delta}, \quad \mathcal{D}_{MM} = -\frac{(1+2\Lambda)}{\tau_\delta}. \quad \text{(AA)}$$

Bogdanov-Takens Bifurcation

At the Bogdanov-Takens bifurcation, the Jacobian has two zero eigenvalues. This means that $\boldsymbol{J}$ has left- and right-eigenvectors which not only satisfy $\boldsymbol{lJ} = \boldsymbol{0}$ and $\boldsymbol{Jr} = \boldsymbol{0}$ but also the orthogonality condition $\boldsymbol{lr} = \boldsymbol{0}$. Denoting the elements of the right-eigenvector as $\boldsymbol{r} = (r_{a1}, ..., r_{aK}, r_\sigma, r_1, ..., r_M)^T$, the right-eigenvector equations at the BT bifurcation are

$$-\frac{r_{ai}}{\tau_i} + \frac{r_\sigma}{\tau_i}\frac{\mathrm{d}a_{i,\infty}}{\mathrm{d}v_\sigma} = 0,$$
$$\sum_{i=1}^{K} r_{ai}\frac{\partial f_\sigma}{\partial a_i} + r_\sigma\left(\frac{\partial f_{\mathrm{S}}}{\partial v_\sigma} - \frac{\sqrt{\Lambda}\rho G_\sigma}{C_\sigma}\right) + r_1\frac{\sqrt{\Lambda}\rho G_\sigma}{C_\sigma} = 0,$$
$$r_{k-1}\frac{\Lambda}{\tau_\delta} - r_k\frac{1+2\Lambda}{\tau_\delta} + r_{k+1}\frac{\Lambda}{\tau_\delta} = 0, \quad k = 1, ..., M-1,$$
$$2r_{M-1}\frac{\Lambda}{\tau_\delta} - r_M\frac{1+2\Lambda}{\tau_\delta} = 0. \quad \text{(AB)}$$

The aim here is to rewrite all the elements of the eigenvector $\boldsymbol{r}$ in terms of r_σ, and we can immediately see that $r_{ai} = r_\sigma \mathrm{d}a_{i,\infty}/\mathrm{d}v_\sigma$. The dendritic elements meanwhile form a discrete difference equation (DDE)

$$r_{k-1}\Lambda - r_k(1+2\Lambda) + r_{k+1}\Lambda = 0, \quad r_0 = r_\sigma, \quad 2r_{M-1}\Lambda - r_M(1+2\Lambda) = 0, \quad \text{(AC)}$$

which has the general solution

$$r_k = c_+\mu_+^k + c_-\mu_-^k, \quad \mu_\pm = \frac{1+2\Lambda \pm \sqrt{1+4\Lambda}}{2\Lambda}. \quad \text{(AD)}$$

Utilising the relations $\mu_+ + \mu_- = (1+2\Lambda)/\Lambda$ and $\mu_+\mu_- = 1$, we can substitute in the boundary conditions to give the specific solution as

$$r_k = r_\sigma\frac{\mu_+^{M-k} + \mu_-^{M-k}}{\mu_+^M + \mu_-^M} \quad \text{(AE)}$$

and hence we have shown that all the right-eigenvector elements can be written in terms of r_σ. Meanwhile the left-eigenvector equations are

$$-\frac{l_{ai}}{\tau_i} + l_\sigma\frac{\partial f_\sigma}{\partial a_i} = 0,$$
$$\sum_{i=1}^{K}\frac{l_{ai}}{\tau_i}\frac{\mathrm{d}a_{i,\infty}}{\mathrm{d}v_\sigma} + l_\sigma\left(\frac{\partial f_{\mathrm{S}}}{\partial v_\sigma} - \frac{\sqrt{\Lambda}\rho G_\sigma}{C_\sigma}\right) + l_1\frac{\partial f_1}{\partial v_\sigma} = 0, \quad l_\sigma\frac{\sqrt{\Lambda}\rho G_\sigma}{C_\sigma} - l_1\frac{1+2\Lambda}{\tau_\delta} + l_2\frac{\Lambda}{\tau_\delta} = 0,$$
$$l_{k-1}\frac{\Lambda}{\tau_\delta} - l_k\frac{1+2\Lambda}{\tau_\delta} + l_{k+1}\frac{\Lambda}{\tau_\delta} = 0, \quad k = 2, ..., M-2,$$
$$l_{M-2}\frac{\Lambda}{\tau_\delta} - l_{M-1}\frac{1+2\Lambda}{\tau_\delta} + l_M\frac{2\Lambda}{\tau_\delta} = 0, \quad l_{M-1}\frac{\Lambda}{\tau_\delta} - l_M\frac{1+2\Lambda}{\tau_\delta} = 0, \quad \text{(AF)}$$

where we can see that $l_{ai} = l_\sigma\tau_i\partial f_\sigma/\partial a_i$. The dendritic elements of $\boldsymbol{l}$ follow the same DDE as before, but with different boundary conditions. This has the solution in terms of l_1

$$l_k = l_1\frac{\mu_+^{M-k} + \mu_-^{M-k}}{\mu_+^{M-1} + \mu_-^{M-1}} \quad \text{(AG)}$$

where $\mu_\pm$ is given by Eq AD and $l_M = l_{M-1}\Lambda/(1+2\Lambda)$. The dendritic elements can be written in terms of l_σ via

$$l_\sigma \frac{\tau_\delta \rho G_\sigma \sqrt{\Lambda}}{C_\sigma} = l_1(1+2\Lambda-\Lambda S), \quad S = l_{k=2}/l_1, \tag{AH}$$

however ρ is intrinsically linked to g_{in}, our bifurcation parameter. We can write ρ in terms of the somatic fixed-point voltage v_σ^* from the somatic right-eigenvector equation

$$\begin{aligned} \sum_{i=1}^{K} r_{ai}\frac{\partial f_\sigma}{\partial a_i} + r_\sigma \frac{\partial f_{\text{S}}}{\partial v_\sigma} - \frac{\rho G_\sigma \sqrt{\Lambda}}{C_\sigma}(r_\sigma - r_1) = 0 \\ \rho(v_\sigma^*) = \frac{C_\sigma r_\sigma}{G_\sigma\sqrt{\Lambda}(r_\sigma - r_1)}\left(\frac{\partial f_{\text{S}}}{\partial v_\sigma} + \sum_{i=1}^{K}\frac{\mathrm{d}a_{i,\infty}}{\mathrm{d}v_\sigma}\frac{\partial f_\sigma}{\partial a_i}\right). \end{aligned} \tag{AI}$$

This means that each term in our eigenvector product is proportional to $l_\sigma r_\sigma$

$$\begin{aligned} \boldsymbol{lr} &= \sum_{i=1}^{K} l_{ai}r_{ai} + l_\sigma r_\sigma + \sum_{k=1}^{M} l_k r_k = 0 \\ &= 1 + \sum_{i=1}^{K}(\tau_i + \alpha_0\tau_\delta)\frac{\partial f_\sigma}{\partial a_i}\frac{\mathrm{d}a_{i,\infty}}{\mathrm{d}v_\sigma} + \alpha_0\tau_\delta\frac{\partial f_{\text{S}}}{\partial v_\sigma}, \end{aligned} \tag{AJ}$$

$$\alpha_0 = \frac{r_\sigma \Sigma_\delta}{(r_\sigma - r_1)(1+2\Lambda-\Lambda S)}, \tag{AK}$$

where Σ_δ represents the summation of the dendritic terms,

$$\Sigma_\delta = \sum_{k=1}^{M-1}\frac{(\mu_+^{M-k}+\mu_-^{M-k})^2}{(\mu_+^M+\mu_-^M)(\mu_+^{M-1}+\mu_-^{M-1})} + \frac{2}{(\mu_+^M+\mu_-^M)(\mu_+^{M-1}+\mu_-^{M-1})}. \tag{AL}$$

Hence for the *discrete* system, one can numerically solve the eigenvector product, Eq AJ, to obtain v_σ^{BT} and then obtain $G_{\text{in}}^{\text{BT}}$ and $I_{\text{ext}}^{\text{BT}}$ from $I'_\infty = 0$ and $I_\infty = 0$ respectively. However, we can also take the continuum limit to obtain a BT equation for a spatially *continuous* system.

A semi-infinite cable attached to a soma can be analysed by taking the limit $M \to \infty$. Note that we will first consider the spatial step size to be still non-zero, $\Delta x > 0$, and later we will take the continuum limit $\Delta x \to 0$. With an infinite number of compartments, the DDEs for r_k and l_k simply greatly as the new distal boundary conditions are

$$\lim_{k\to\infty}|r_k| < \infty, \quad \lim_{k\to\infty}|l_k| < \infty. \tag{AM}$$

From our earlier definition of $\mu_\pm$, for $\Lambda > 0$ $|\mu_+| > 1$ and $|\mu_-| < 1$. This for both l_k and r_k the solutions are

$$r_k = r_\sigma \mu_-^k, \quad l_k = l_1\mu_-^{k-1}, \quad k = 1, 2, ... \tag{AN}$$

This means that the summation of dendritic terms is

$$\Sigma_\delta = \sum_{k=1}^{\infty}\mu_-^{2k-1} = \frac{\mu_-}{1-\mu_-^2}, \tag{AO}$$

and hence the factor α_0 is

$$\alpha_0 = \frac{\mu_-}{(1-\mu_-)(1+2\Lambda-\Lambda\mu_-)(1-\mu_-^2)}. \tag{AP}$$

Now we take the continuum limit $\Delta x \to 0$ which in this case is equivalent to $\Lambda \to \infty$. α_0 is the only constant that depends on Λ, and has the limit

$$\lim_{\Lambda\to\infty} \alpha_0 = \frac{1}{2}. \tag{AQ}$$

Substituting α_0 into the BT condition thus gives

$$\sum_{i=1}^{K} \left(\frac{\tau_\delta}{2} + \tau_i\right) \frac{\partial f_\sigma}{\partial a_i} \frac{\mathrm{d}a_{i,\infty}}{\mathrm{d}v_\sigma} + 1 + \frac{\tau_\delta}{2} \frac{\partial f_\mathrm{S}}{\partial v_\sigma} = 0. \tag{AR}$$

For the finite cable, after resolving the summation (Eq AL), we define $\Delta z = \Delta x/\lambda$. This allows M to be rewritten as $M = \ell/\Delta z$ and $\Lambda = \Delta z^{-2}$. After much algebra, taking the limit $\Delta z \to 0$ gives for α_0

$$\lim_{\Delta z\to 0} \alpha_0 = \frac{1}{2} + \frac{\ell}{2\cosh\ell \sinh\ell}. \tag{AS}$$

Hopf Bifurcation

At the Hopf bifurcation, there exist right-eigenvectors of the Jacobian $\boldsymbol{J}$ which satisfy $\boldsymbol{J}\boldsymbol{q} = i\omega_\mathrm{H}\boldsymbol{q}$ and $\boldsymbol{J}\overline{\boldsymbol{q}} = -i\omega_\mathrm{H}\overline{\boldsymbol{q}}$. We can use the discretised Jacobian outlined earlier to obtain an expressing for the somatic voltage at the Hopf bifurcation, v_σ^H. For the right eigenvectors we will use the indexing $\boldsymbol{q} = (q_{a1}, ..., q_{aK}, q_\sigma, q_1, ..., q_M)$, and hence our right-eigenvector equations are

$$-\frac{q_{ai}}{\tau_i} + \frac{q_\sigma}{\tau_i}\frac{\mathrm{d}a_{i,\infty}}{\mathrm{d}v_\sigma} = i\omega_\mathrm{H} q_{ai}, \quad \sum_{i=1}^{K} q_{ai}\frac{\partial f_\sigma}{\partial a_i} + q_\sigma\left(\frac{\partial f_\mathrm{S}}{\partial v_\sigma} - \frac{\sqrt{\Lambda}\rho G_\sigma}{C_\sigma}\right) + q_1\frac{\partial f_\sigma}{\partial v_1} = i\omega_\mathrm{H} q_\sigma,$$
$$q_{k-1}\frac{\Lambda}{\tau_\delta} - q_k\frac{1+2\Lambda}{\tau_\delta} + q_{k+1}\frac{\Lambda}{\tau_\delta} = i\omega_\mathrm{H} q_k, \quad k = 1, ..., M-1, \quad 2q_{M-1}\frac{\Lambda}{\tau_\delta} - q_M\frac{1+2\Lambda}{\tau_\delta} = i\omega q_M. \tag{AT}$$

We see that the dendritic elements follow a DDE equation similar to the one for the BT bifurcation. We rewrite the DDE and its boundary conditions as

$$\begin{aligned} q_{k-1}\Lambda - q_k(1 + 2\Lambda + i\omega_\mathrm{H}\tau_\delta) + q_{k+1}\Lambda = 0, \quad q_0 = q_\sigma, \\ 2q_{M-1}\Lambda - q_M(1 + 2\Lambda + i\omega_\mathrm{H}\tau_\delta) = 0, \end{aligned} \tag{AU}$$

where the DDE has a general solution in terms of the bases $\eta_\pm$, a complex analogue of $\mu_\pm$

$$q_k = c_+\eta_+^k + c_-\eta_-^k, \quad \eta_\pm = \frac{1 + 2\Lambda + i\omega_\mathrm{H}\tau_\delta \pm \sqrt{(1 + 2\Lambda + i\omega_\mathrm{H}\tau_\delta)^2 - 4\Lambda^2}}{2\Lambda}, \tag{AV}$$

for which the properties $\eta_+\eta_- = 1$ and $\eta_+ + \eta_- = (1 + 2\Lambda + i\omega\tau_\delta)/\Lambda$ hold. We use the boundary conditions to find $c_\pm$ and hence the specific solution to q_k

$$q_k = q_\sigma \frac{\eta_+^{M-k} + \eta_-^{M-k}}{\eta_+^M + \eta_-^M}. \tag{AW}$$

Substituting q_1 and q_{ai} into the equation for q_σ yields

$$\sum_{i=1}^{K} \frac{1 - i\omega_\mathrm{H}\tau_i}{1 + \omega_\mathrm{H}^2\tau_i^2}\frac{\partial f_\sigma}{\partial a_i}\frac{\mathrm{d}a_{i,\infty}}{\mathrm{d}v_\sigma} + \frac{\partial f_\mathrm{S}}{\partial v_\sigma} + \frac{\rho G_\sigma\sqrt{\Lambda}}{C_\sigma}\left[\frac{\eta_+^{M-1} + \eta_-^{M-1}}{\eta_+^M + \eta_-^M} - 1\right] - i\omega_\mathrm{H} = 0. \tag{AX}$$

Turning back to the eigenvector $\overline{\boldsymbol{q}}$, we obtain the same form of specific solution for the dendritic eigenvector elements, but this time in terms of $\overline{\eta}_\pm$

$$\overline{\eta}_\pm = \frac{1 + 2\Lambda - i\omega_\mathrm{H}\tau_\delta \pm \sqrt{(1 + 2\Lambda - i\omega_\mathrm{H}\tau_\delta)^2 - 4\Lambda^2}}{2\Lambda}. \tag{AY}$$

This means that the same substitutions into the equation for $\overline{q}_\sigma$ gives

$$\sum_{i=1}^{K} \frac{1+i\omega_{\mathrm{H}}\tau_i}{1+\omega_{\mathrm{H}}^2\tau_i^2}\frac{\partial f_\sigma}{\partial a_i}\frac{\mathrm{d}a_{i,\infty}}{\mathrm{d}v_\sigma} + \frac{\partial f_{\mathrm{S}}}{\partial v_\sigma} + \frac{\rho G_\sigma\sqrt{\Lambda}}{C_\sigma}\left[\frac{\overline{\eta}_+^{M-1}+\overline{\eta}_-^{M-1}}{\overline{\eta}_+^{M}+\overline{\eta}_-^{M}} - 1\right] + i\omega_{\mathrm{H}} = 0. \tag{AZ}$$

For the semi-infinite dendrite first we take $M \to \infty$, which has the effect of simplifying the specific solution of the DDEs to

$$q_k = q_\sigma \eta_-^k, \quad \overline{q}_k = q_\sigma \overline{\eta}_-^k. \tag{BA}$$

The equations for q_σ and $\overline{q}_\sigma$ are hence simplified to

$$\sum_{i=1}^{K} \frac{1-i\omega_{\mathrm{H}}\tau_i}{1+\omega_{\mathrm{H}}^2\tau_i^2}\frac{\partial f_\sigma}{\partial a_i}\frac{\mathrm{d}a_{i,\infty}}{\mathrm{d}v_\sigma} + \frac{\partial f_{\mathrm{S}}}{\partial v_\sigma} + \frac{\rho G_\sigma\sqrt{\Lambda}}{C_\sigma}(\eta_- - 1) - i\omega_{\mathrm{H}} = 0, \tag{BB}$$

$$\sum_{i=1}^{K} \frac{1+i\omega_{\mathrm{H}}\tau_i}{1+\omega_{\mathrm{H}}^2\tau_i^2}\frac{\partial f_\sigma}{\partial a_i}\frac{\mathrm{d}a_{i,\infty}}{\mathrm{d}v_\sigma} + \frac{\partial f_{\mathrm{S}}}{\partial v_\sigma} + \frac{\rho G_\sigma\sqrt{\Lambda}}{C_\sigma}(\overline{\eta}_- - 1) + i\omega_{\mathrm{H}} = 0. \tag{BC}$$

Now we take the continuum limit ($\Lambda \to \infty$) of the only term that varies with Λ

$$\lim_{\Lambda\to\infty} \sqrt{\Lambda}(\eta_- - 1) = -\frac{\sqrt{4\Lambda + 4i\omega_{\mathrm{H}}\tau_\delta\Lambda}}{2\sqrt{\Lambda}} = -\sqrt{1+i\omega_{\mathrm{H}}\tau_\delta} = -\gamma(\omega_{\mathrm{H}}). \tag{BD}$$

We similarly find that

$$\lim_{\Lambda\to\infty} \sqrt{\Lambda}(\overline{\eta} - 1) = -\sqrt{1-i\omega_{\mathrm{H}}\tau_\delta} = -\gamma(-\omega_{\mathrm{H}}). \tag{BE}$$

It can be shown that $\gamma(\omega_{\mathrm{H}})$ and $\gamma(-\omega_{\mathrm{H}})$ are complex conjugates with a sum and difference given by

$$\gamma(\omega_{\mathrm{H}}) + \gamma(-\omega_{\mathrm{H}}) := s_\gamma(\omega_{\mathrm{H}}) = \sqrt{2+2\sqrt{1+\omega_{\mathrm{H}}^2\tau_\delta^2}}, \tag{BF}$$

$$\gamma(\omega_{\mathrm{H}}) - \gamma(-\omega_{\mathrm{H}}) := id_\gamma(\omega_{\mathrm{H}}) = i\,\mathrm{sgn}(\omega_{\mathrm{H}})\sqrt{-2+2\sqrt{1+\omega_{\mathrm{H}}^2\tau_\delta^2}}. \tag{BG}$$

This means that summing Eqs BB and BC in the continuum limit gives the real-valued equation

$$\sum_{i=1}^{K} \frac{1}{1+\omega_{\mathrm{H}}^2\tau_i^2}\frac{\partial f_\sigma}{\partial a_i}\frac{\mathrm{d}a_{i,\infty}}{\mathrm{d}v_\sigma} + \frac{\partial f_{\mathrm{S}}}{\partial v_\sigma} - \frac{\rho G_\sigma}{2C_\sigma}s_\gamma(\omega_{\mathrm{H}}) = 0, \tag{BH}$$

while subtracting Eq BB from Eq BC gives an equation in terms of the imaginary part

$$\sum_{i=1}^{K} \frac{\tau_i}{1+\omega_{\mathrm{H}}^2\tau_i^2}\frac{\partial f_\sigma}{\partial a_i}\frac{\mathrm{d}a_{i,\infty}}{\mathrm{d}v_\sigma} + \frac{\rho G_\sigma}{2C_\sigma}\frac{d_\gamma(\omega_{\mathrm{H}})}{\omega_{\mathrm{H}}} + 1 = 0. \tag{BI}$$

These nonlinear equations can be solved simultaneously to find ω_{H} and v_σ^{H}.

To find the finite continuum limit solution for the Hopf bifurcation, we first find the continuum limits of

$$\sqrt{\Lambda}\left[\frac{\eta_+^{M-1}+\eta_-^{M-1}}{\eta_+^{M}+\eta_-^{M}} - 1\right] \quad \text{and} \quad \sqrt{\Lambda}\left[\frac{\overline{\eta}_+^{M-1}+\overline{\eta}_-^{M-1}}{\overline{\eta}_+^{M}+\overline{\eta}_-^{M}} - 1\right] \tag{BJ}$$

in Eqs AX and AZ by substituting $\Lambda = \Delta z^{-2}$ and $M = l/\Delta z$ as in the BT section. After doing so, the first Hopf equation (Eq AX) becomes

$$\sum_{i=1}^{K} \frac{1-i\omega_{\mathrm{H}}\tau_i}{1+\omega_{\mathrm{H}}^2\tau_i^2}\frac{\partial f_\sigma}{\partial a_i}\frac{\mathrm{d}a_{i,\infty}}{\mathrm{d}v_\sigma} + \frac{\partial f_{\mathrm{S}}}{\partial v_\sigma} - \frac{\rho G_\sigma}{C_\sigma}\gamma(\omega_{\mathrm{H}})\tanh[l\gamma(\omega_{\mathrm{H}})] - i\omega_{\mathrm{H}} = 0, \tag{BK}$$

and the continuum-limit of the second Hopf equation (Eq AZ) is

$$\sum_{i=1}^{K}\frac{1+i\omega_{\mathrm{H}}\tau_i}{1+\omega_{\mathrm{H}}^2\tau_i^2}\frac{\partial f_\sigma}{\partial a_i}\frac{\mathrm{d}a_{i,\infty}}{\mathrm{d}v_\sigma}+\frac{\partial f_{\mathrm{S}}}{\partial v_\sigma}-\frac{\rho G_\sigma}{C_\sigma}\gamma(-\omega_{\mathrm{H}})\tanh[l\gamma(-\omega_{\mathrm{H}})]+i\omega_{\mathrm{H}}=0. \tag{BL}$$

Now when we sum and difference, Eqs BK and BL, we get equations for the real and imaginary parts

$$\sum_{i=1}^{K}\frac{1}{1+\omega_{\mathrm{H}}^2\tau_i^2}\frac{\partial f_\sigma}{\partial a_i}\frac{\mathrm{d}a_{i,\infty}}{\partial v_\sigma}+\frac{\partial f_{\mathrm{S}}}{\partial v_\sigma}-\frac{\rho G_\sigma}{2C_\sigma}\frac{s_\gamma\sinh(ls_\gamma)-d_\gamma\sin(ld_\gamma)}{\cosh(ls_\gamma)+\cos(ld_\gamma)}=0, \tag{BM}$$

$$\sum_{i=1}^{K}\frac{\tau_i}{1+\omega_{\mathrm{H}}^2\tau_i^2}\frac{\partial f_\sigma}{\partial a_i}\frac{\mathrm{d}a_{i,\infty}}{\mathrm{d}v_\sigma}+\frac{\rho G_\sigma}{2C_\sigma}\frac{d_\gamma\sinh(ls_\gamma)+s_\gamma\sin(ld_\gamma)}{\omega_{\mathrm{H}}[\cosh(ls_\gamma)+\cos(ld_\gamma)]}+1=0. \tag{BN}$$

These two nonlinear equations must be solved simultaneously. In this case, we used a trust-region method implemented by the NLsolve.jl package [6].

Criticality of the Hopf Bifurcation

The criticality of the Hopf bifurcation can be obtained from the first Lyapunov coefficient of the equilibrium that undergoes the Hopf bifurcation

$$\mathcal{L}_1=\frac{1}{2\omega_{\mathrm{H}}}\mathrm{Re}\left\{\overline{\boldsymbol{p}}\boldsymbol{C}(\boldsymbol{q},\boldsymbol{q},\overline{\boldsymbol{q}})-2\overline{\boldsymbol{p}}\boldsymbol{B}[\boldsymbol{q},\boldsymbol{J}^{-1}\boldsymbol{B}(\boldsymbol{q},\overline{\boldsymbol{q}})]+\overline{\boldsymbol{p}}\boldsymbol{B}[\overline{\boldsymbol{q}},(2i\omega_{\mathrm{H}}\boldsymbol{I}-\boldsymbol{J})^{-1}\boldsymbol{B}(\boldsymbol{q},\boldsymbol{q})]\right\}, \tag{BO}$$

where $\boldsymbol{q}$ is the right-eigenvector of $\boldsymbol{J}$ at the Hopf bifurcation found earlier, $\boldsymbol{p}$ is the left-eigenvector of $\boldsymbol{J}$, and $\boldsymbol{B}$ and $\boldsymbol{C}$ are the second and third order tensors of the system. When $\mathcal{L}_1<0$, the Hopf bifurcation is supercritical, while when $\mathcal{L}_1>0$ it is subcritical. The left-eigenvectors satisfy $\boldsymbol{p}\boldsymbol{J}=-i\omega_{\mathrm{H}}\boldsymbol{p}$ and $\overline{\boldsymbol{p}}\boldsymbol{J}=i\omega_{\mathrm{H}}\overline{\boldsymbol{p}}$ while the ith element of each evaluated tensor is

$$B_i(\boldsymbol{x},\boldsymbol{y})=\sum_{j,k}\frac{\partial^2 f_i}{\partial\xi_j\partial\xi_k}x_jy_k,\quad C_i(\boldsymbol{x},\boldsymbol{y},\boldsymbol{z})=\sum_{j,k,l}\frac{\partial^3 f_i}{\partial\xi_j\partial\xi_k\partial\xi_l}x_jy_kz_l. \tag{BP}$$

Due to the linearity of the passive dendrite, the dendritic terms of B_i and C_i will be zero. Thus, we only need calculate the $K+1$ terms for each active value and the somatic voltage, which use the eigenvector elements $q_i,...,q_K,q_\sigma$. Using the normalisation $\overline{\boldsymbol{p}}\boldsymbol{q}=1$, we can write all the eigenvector elements of $\boldsymbol{p}$ and $\boldsymbol{q}$ in terms of q_σ in the continuum limit

$$\overline{p}_\sigma=\frac{q_\sigma}{\kappa},\quad \kappa=\sum_{i=1}^{K}\frac{\tau_i}{(1+i\omega_{\mathrm{H}}\tau_i)^2}\frac{\partial f_\sigma}{\partial a_i}\frac{\mathrm{d}a_{i,\infty}}{\mathrm{d}v_\sigma}+1+\frac{\tau_\delta\rho g_\sigma}{C_\sigma}\frac{(e^{2\ell\gamma}-e^{-2\ell\gamma}+4\ell\gamma)}{2\gamma(e^{\ell\gamma}+e^{-\ell\gamma})^2}, \tag{BQ}$$

$$\overline{p}_{ai}=\frac{\overline{p}_\sigma\tau_i}{1+i\omega_{\mathrm{H}}\tau_i}\frac{\partial f_\sigma}{\partial a_i},\quad q_{ai}=\frac{q_\sigma}{1+i\omega_{\mathrm{H}}\tau_i}\frac{\mathrm{d}a_{i,\infty}}{\mathrm{d}v_\sigma}. \tag{BR}$$

For the terms involving inverse matrices, elements required for calculation of $\boldsymbol{B}$ also have continuum limits. Denoting $\boldsymbol{u}=\boldsymbol{J}^{-1}\boldsymbol{B}(\boldsymbol{q},\overline{\boldsymbol{q}})$, we require the elements $u_{a1},...,u_{aK},u_\sigma$. Since only the first $K+1$ elements of $\boldsymbol{B}$ are non-zero, this means only the calculation of $(K+1)\times(K+1)$ elements of $\boldsymbol{J}^{-1}$ is necessary. The block structure of $\boldsymbol{J}$ defined earlier (Eq R) means that its inverse elements can be found in terms of the blocks

$$\boldsymbol{J}=\begin{pmatrix}\mathcal{A} & \mathcal{B}\\ \mathcal{C} & \mathcal{D}\end{pmatrix},\quad \boldsymbol{J}^{-1}=\begin{pmatrix}\mathcal{A}^{-1}+\mathcal{A}^{-1}\mathcal{B}\mathcal{S}^{-1}\mathcal{C}\mathcal{A}^{-1} & -\mathcal{A}^{-1}\mathcal{B}\mathcal{S}^{-1}\\ -\mathcal{S}^{-1}\mathcal{C}\mathcal{A}^{-1} & \mathcal{S}^{-1}\end{pmatrix}. \tag{BS}$$

Here $\mathcal{S}=\mathcal{D}-\mathcal{C}\mathcal{A}^{-1}\mathcal{B}$ is the Schur complement. Using this approach, the upper-left $(K+1)\times(K+1)$ quadrant of $\boldsymbol{J}^{-1}$ can be found as

$$[\boldsymbol{J}^{-1}]_{ij}=\begin{cases}-\tau_i+[\mathcal{S}^{-1}]_{11}\tau_i\frac{\mathrm{d}a_{i,\infty}}{\mathrm{d}v_\sigma}\frac{\partial f_\sigma}{\partial a_i}, & i=j,\quad i,j\neq\sigma\\ [\mathcal{S}^{-1}]_{11}\tau_j\frac{\mathrm{d}a_{i,\infty}}{\mathrm{d}v_\sigma}\frac{\partial f_\sigma}{\partial a_j}, & i\neq j,\quad i,j\neq\sigma\\ [\mathcal{S}^{-1}]_{11}\frac{\mathrm{d}a_{i,\infty}}{\mathrm{d}v_\sigma}, & i\neq\sigma,\quad j=\sigma\\ [\mathcal{S}^{-1}]_{11}\tau_j\frac{\partial f_\sigma}{\partial a_j}, & i=\sigma,\quad j\neq\sigma\\ [\mathcal{S}^{-1}]_{11}, & i=j=\sigma\end{cases}, \tag{BT}$$

where in the continuum limit $[\mathcal{S}^{-1}]_{11}$ is the inverse of the total derivative of the somatic voltage equation with respect to v_σ

$$[\mathcal{S}^{-1}]_{11} = \left(\frac{\partial f_{\rm S}}{\partial v_\sigma} + \sum_{i=1}^{K} \tau_i \frac{\partial f_{ai}}{\partial v_\sigma} \frac{\partial f_\sigma}{\partial a_i} - \frac{\rho G_\sigma}{C_\sigma} \tanh \ell \right)^{-1} . \tag{BU}$$

The $(K+1) \times (K+1)$ upper-left quadrant can be calculated for the inverse of $\tilde{\boldsymbol{J}} = \boldsymbol{J} - 2i\omega_{\rm H} \boldsymbol{I}$ in a similar manner, yielding

$$[\tilde{\boldsymbol{J}}^{-1}]_{ij} = \begin{cases} -\tilde{\tau}_i + [\tilde{\mathcal{S}}^{-1}]_{11} \frac{\tilde{\tau}_i^2}{\tau_i} \frac{{\rm d}a_{i,\infty}}{{\rm d}v_\sigma} \frac{\partial f_\sigma}{\partial a_i}, \quad i = j, \quad i,j \neq \sigma \\ [\tilde{\mathcal{S}}^{-1}]_{11} \frac{\tilde{\tau}_i \tilde{\tau}_j}{\tau_i} \frac{{\rm d}a_{i,\infty}}{{\rm d}v_\sigma} \frac{\partial f_\sigma}{\partial a_j}, \quad i \neq j, \quad i,j \neq \sigma \\ [\tilde{\mathcal{S}}^{-1}]_{11} \frac{\tilde{\tau}_i}{\tau_i} \frac{{\rm d}a_{i,\infty}}{{\rm d}v_\sigma}, \quad i \neq \sigma, \quad j = \sigma \\ [\tilde{\mathcal{S}}^{-1}]_{11} \tilde{\tau}_j \frac{\partial f_\sigma}{\partial a_j}, \quad i = \sigma, \quad j \neq \sigma \\ [\tilde{\mathcal{S}}^{-1}]_{11}, \quad i = j = \sigma \end{cases} , \tag{BV}$$

where $\tilde{\tau}_i = \tau_i / (1 + 2i\omega_{\rm H} \tau_i)$ and

$$[\tilde{\mathcal{S}}^{-1}]_{11} = \left\{ \frac{\partial f_{\rm S}}{\partial v_\sigma} + \sum_{i=1}^{K} \tilde{\tau}_i \frac{\partial f_\sigma}{\partial a_i} \frac{\partial f_{ai}}{\partial v_\sigma} - 2i\omega_{\rm H} - \frac{\rho G_\sigma}{C_\sigma} \gamma(2\omega_{\rm H}) \tanh[\ell \gamma(2\omega_{\rm H})] \right\}^{-1} , \tag{BW}$$

with γ having the same form as before. This provides us with all the information required to calculate the criticality of a Hopf bifurcation in the continuum limit.

Dendritic Current Input

Moving the external current input to an arbitrary dendritic location $x_{\rm in}$ changes the cable equation to

$$\tau_\delta \frac{\partial v_\delta}{\partial t} = E_L - v_\delta + \lambda^2 \frac{\partial^2 v_\delta}{\partial x^2} + \frac{\lambda I_{\rm ext}}{\rho G_\sigma} \delta(x - x_{\rm in}), \tag{BX}$$

while the somatic equation remains as in (16) with $I_{\rm ext} = 0$ at the soma. Setting all time derivatives to zero, the dendritic equilibrium voltage for the semi-infinite dendrite satisfies

$$v_\delta^*(x) = E_L + (v_\sigma^* - E_L) e^{-x/\lambda} + [e^{-|x - x_{\rm in}|/\lambda} - e^{-|x - x_{\rm in}|/\lambda}], \tag{BY}$$

which means that the somatic fixed point potential is found by solving

$$I_\infty(v_\sigma) = C_\sigma f_{\rm S}(\boldsymbol{a}_\infty(v_\sigma), v_\sigma) + \rho G_\sigma (E_L - v_\sigma) + I_{\rm ext} e^{-x_{\rm in}/\lambda} = 0. \tag{BZ}$$

Meanwhile for finite dendrite, the dendritic equilibrium voltage with $z = x/\lambda$ obeys

$$\begin{aligned} v_\delta^*(x) = E_L + (v_\sigma - E_L) \cosh z + \left\{ \frac{I_{\rm ext}}{\rho G_\sigma} \frac{\cosh(\ell - z)}{\cosh \ell} - (v_\sigma - E_L) \tanh \ell \right\} \sinh z \\ -\theta(z - z_{\rm in}) \frac{I_{\rm ext}}{\rho G_\sigma} \sinh(z - z_{\rm in}), \end{aligned} \tag{CA}$$

which when substituted into the somatic boundary condition yields

$$I_\infty(v_\sigma) = C_\sigma f_{\rm S}(\boldsymbol{a}_\infty(v_\sigma), v_\sigma) + \rho G_\sigma \tanh \ell (E_L - v_\sigma) + I_{\rm ext} \frac{\cosh(\ell - z_{\rm in})}{\cosh \ell} = 0. \tag{CB}$$

In both the semi-infinite and finite models, the only term of the steady-state current equations affected by the spatial separation between the soma at point of current injection, $x_{\rm in}$, is the external current term. The steady-state current equations converge to the somatically driven case when $x_{\rm in} = 0$ as expected.

This means that for the saddle-node and cusp bifurcations, differentiating Eq BZ or Eq CB with respect to v_σ removes all dependence on the location of synaptic input. Therefore, for the same values of

τ_δ and ρ, v_σ^{SN} does not vary with x_{in} and has the same value as when external current is applied at the soma. Similarly, for the same τ_δ, ρ^{C} and v^{C} do not depend on x_{in}. $I_{\mathrm{ext}}^{\mathrm{SN}}$ and $I_{\mathrm{ext}}^{\mathrm{C}}$ are the only parameters altered by the spatial location.

For the BT and Hopf bifurcations, we must discretise the cable equation and perform the method of lines. However, since the external dendritic drive does not depend on any of the system variables, the discretised Jacobian $\boldsymbol{J}$ will not depend x_{in} and will thus be identical to the somatically driven scenario. Since the bifurcation parameters for the BT and Hopf bifurcations are derived from the eigenvectors of $\boldsymbol{J}$, this means that they have values which do not vary with x_{in}. Only I_{ext} varies with x_{in}, as was the case for the saddle-node and cusp bifurcations.

Estimation of Global Bifurcations

Global bifurcations govern many spiking onset types and spiking onset transitions, but cannot be directly calculated from the properties of fixed points. Therefore one must use numerical continuation methods [7, 8] and/or numerical simulation in order to estimate the locations of global bifurcations. For simplicity of implementation and to be understood by a wider audience, here we outline how simulation can be used to estimate the location of two global bifurcations of interest.

Homoclinic Bifurcation

At a homoclinic (HOM) bifurcation, a homoclinic orbit is formed at a saddle. If this orbit is stable, then it forms a spiking cycle. HOM onset is class I but its f–I curve typically grows very rapidly in comparison to SNIC onset [9]. Since the HOM bifurcation does not involve changes to the stability of fixed points, a stable homoclinic orbit typically coexists with a stable fixed point. This means that HOM onset allows bistability between quiescence and regular spiking. If the homoclinic orbit contains a single fixed point (other than the saddle), then it is termed a small-HOM (sHOM) bifurcation, while if orbit contains all three fixed points, it is a big-HOM (bHOM) bifurcation.

Using I_{ext} as a bifurcation parameter, if a stable HOM bifurcation exists then it often precedes a SN bifurcation, $I_{\mathrm{ext}}^{\mathrm{HOM}} < I_{\mathrm{ext}}^{\mathrm{SN}}$. Thus we can find the HOM bifurcation as follows:

1. At a given $G_{\mathrm{in}} < G_{\mathrm{in}}^{\mathrm{BT}}$, find the onset current $I_{\mathrm{ext}}^{\mathrm{on}}$ for regular spiking.
2. If $I_{\mathrm{ext}}^{\mathrm{on}} > I_{\mathrm{ext}}^{\mathrm{SN}}$ there is no bistability, and spiking onset occurs via a SNIC bifurcation.
3. If $I_{\mathrm{ext}}^{\mathrm{on}} < I_{\mathrm{ext}}^{\mathrm{SN}}$, bistability exists and a HOM bifurcation is a candidate for the onset type.
4. Repeat this process at various G_{in} to obtain an estimate of the HOM bifurcation for the input conductance range of interest.

We specify $G_{\mathrm{in}} < G_{\mathrm{in}}^{\mathrm{BT}}$ in step (1) here to avoid the presence of Hopf bifurcations. In step (3), we can only conclude that a HOM bifurcation is a candidate for the onset type because the true onset bifurcation may be a fold of limit cycles (FLC) that shortly precedes a subcritical HOM bifurcation [10, 3]. Nevertheless, this procedure forms the basis of finding the switch between SNIC and HOM onset.

Saddle-Node-Loop (SNL) Bifurcation

A saddle-node-loop (SNL) bifurcation is where a saddle-node and HOM bifurcation meet and has codimension two. With $(G_{\mathrm{in}}, I_{\mathrm{ext}})$ as bifurcation parameters, we can therefore switch between SNIC and HOM onset by varying G_{in} around this bifurcation. The SNL bifurcation can be estimated via the following bisection procedure:

1. Find input conductances $G_{\mathrm{in}}^{\mathrm{SNIC}}$ and $G_{\mathrm{in}}^{\mathrm{HOM}}$ for which spiking onset occurs via SNIC and (candidate) HOM bifurcations respectively.
2. Choose a new input conductance between these two points $G_{\mathrm{in}}^{\mathrm{test}} = \frac{1}{2}(G_{\mathrm{in}}^{\mathrm{SNIC}} + G_{\mathrm{in}}^{\mathrm{HOM}})$ and find its onset current $I_{\mathrm{ext}}^{\mathrm{test}}$.

3. Using the procedure for evaluating whether the onset is due to a HOM bifurcation, evaluate the candidate bifurcation type for onset at $G_{\mathrm{in}}^{\mathrm{test}}$.

4. Repeat step (1) with $G_{\mathrm{in}}^{\mathrm{test}}$ replacing the bound of its candidate bifurcation type.

5. Stop after a given number of steps or after $G_{\mathrm{in}}^{\mathrm{test}}$ converges. The final value of $G_{\mathrm{in}}^{\mathrm{test}}$ gives an estimate of $G_{\mathrm{in}}^{\mathrm{SNL}}$.

Reconstructed Morphology PRCs

Four different tree "depths" of the reconstructed Purkinje cell were chosen for simulation, where a tree of depth n includes all parts of the tree which lie no more n branching nodes from the soma. Thus a depth of $n = 0$ includes only the soma, a depth of $n = 1$ includes the soma and all sections up to the first branching node, and so on. The four morphological depths chosen in our simulations were $n = 8, 13, 14$ and an unbounded n which includes the whole tree.

The soma was considered as all compartments of the original swc file which were labelled as the soma. The parameters were provided to the somatic and dendritic compartments are given in Table B. All reversal potentials and parameters governing the active variables take the same values as the DS model in Table A. Note that the ratios between g_L in the soma and the active conductance densities g_{Ca}, g_{K} are the same as in the DS model. The capacitances per area in the dendrite have been adjusted such that $g_{L,\sigma}/c_\sigma = 10$ ms and $g_{L,\delta}/c_\delta = 2.5$ ms. These parameters meant that the depths chosen corresponded to dendritic to somatic input conductance ratios of $G_\delta/G_\sigma = (0.424, 1.28, 1.49, 1.93)$.

Parameter	Value	Units
$g_{L,\sigma}$	225	µS/cm^2
$g_{L,\delta}$	50	µS/cm^2
c_σ	2.25	pF
c_σ	0.125	pF
g_{Ca}	$2g_{L,\sigma}$	µS/cm^2
g_{K}	$4g_{L,\sigma}$	µS/cm^2
r_a	250	Ωcm

Table B. Parameters used in the morphologically reconstructed model. The subscripts σ and δ refer to parameter values in somatic and dendritic compartments respectively.

At each depth, the onset current was found by the same method as for the DS model: the lowest value of I_{ext} that achieves a firing rate of at least 1 Hz. PRCs were found at these onset currents by perturbing only the soma to give a maximum phase shift lower than 0.1.

References

[1] Morris C, Lecar H. Voltage oscillations in the barnacle giant muscle fiber. Biophysical journal. 1981;35(1):193-213.

[2] Hesse J, Schleimer JH, Schreiber S. Qualitative changes in phase-response curve and synchronization at the saddle-node-loop bifurcation. Physical Review E. 2017;95(5):052203.

[3] Kirst C, Ammer J, Felmy F, Herz A, Stemmler M. GABA regulates resonance and spike rate encoding via a universal mechanism that underlies the modulation of action potential generation. bioRxiv. 2017:206581.

[4] Al-Darabsah I, Campbell SA. M-current induced Bogdanov–Takens bifurcation and switching of neuron excitability class. The Journal of Mathematical Neuroscience. 2021;11(1):1-26.

[5] Verwer JG, Sanz-Serna JM. Convergence of method of lines approximations to partial differential equations. Computing. 1984;33(3-4):297-313.

[6] JuliaNLSolvers/NLsolve.jl: v4.5.1 (v4.5.1); 2020. Available from: https://doi.org/10.5281/zenodo.4404703.

[7] Doedel EJ, Champneys AR, Fairgrieve T, Kuznetsov Y, Oldeman B, Paffenroth R, et al. Auto-07p: Continuation and bifurcation software for ordinary differential equations (2007). Available for download from http://indy cs concordia ca/auto. 2007.

[8] Allgower EL, Georg K. Numerical continuation methods: an introduction. vol. 13. Springer Science & Business Media; 2012.

[9] Izhikevich EM. Bifurcations. MIT press; 2007.

[10] De Maesschalck P, Wechselberger M. Neural excitability and singular bifurcations. The Journal of Mathematical Neuroscience (JMN). 2015;5(1):1-32.
